# DGT-Based Assessment of Antibiotics and Hormones in a Typical Wastewater Treatment Plant and Its Receiving Water in Shanghai: Implications for Aquaculture Reuse

**DOI:** 10.3390/toxics13110970

**Published:** 2025-11-11

**Authors:** Yin Huang, Zheng Zhang, Chaofeng Sun, Luting Wen, Qian Wang, Yanhao Yang

**Affiliations:** 1 Guangxi Key Laboratory of Aquatic Genetic Breeding and Healthy Aquaculture, Guangxi Institute of Fisheries, Nanning 530021, China; 2 College of Oceanography and Ecological Science, Shanghai Ocean University, Shanghai 201306, China; 3 CTIesting International Group Co., Ltd., Shenzhen 518000, China

**Keywords:** bioavailability, DGT, antibiotics, environmental hormones, wastewater treatment plant, water reuse for aquaculture

## Abstract

Assessment of the environmental behavior of environmental hormones and antibiotics along the processes in typical wastewater treatment plants (WWTPs) based on bioavailable concentrations reflects the negative effects of pollutants from WWTPs on aquatic organisms more directly, as well as the potential for reusing the effluent and receiving waters for aquaculture. This study measured bioavailable concentrations in a typical WWTP and its receiving water body using the XAD-DGT samplers during dry and wet seasons. Firstly, the results confirmed the applicability of XAD-DGT in WWTP and the receiving water. Then, significant season and process-dependent variations were observed. The primary treatment occasionally led to concentration rebound due to desorption during the dry season, secondary treatment exhibited considerable variability depending on the physicochemical properties of the contaminants, and tertiary treatment consistently performed well (>80%). Based on XAD-DGT-measured bioavailable concentrations, the risks posed by environmental hormones and antibiotics in the effluent and receiving water body were determined to assess their potential for aquaculture reuse. The result indicated that the effluent water is applicable for fish aquaculture; however, further removal techniques, like adsorption or advanced oxidation, should be applied to crustacean cultivation, especially for contaminants like environmental hormones. For the water body, it was only feasible for crustacean aquaculture. Pre-treatments based on adsorption, sedimentation, or oxidation processes are necessary to remove environmental hormones and antibiotics if these areas are planned for aquaculture. This study provides an important scientific basis for a more accurate assessment of the environmental behavior of emerging contaminants, reuse directions of WWTP effluent, as well as the corresponding receiving waters.

## 1. Introduction

Shanghai is the largest city in China, with a population of 24.9 million. Its annual wastewater, including domestic and industrial wastewater, is approximately 2.20 × 10^9^ m^3^/year, and 80% of the wastewater is discharged into the Yangtze River and adjacent coastal waters after treatment by wastewater treatment plants (WWTPs) [1]. The effluent is considered a significant freshwater resource, and its recycling is important for alleviating water scarcity and improving environmental quality. Due to the high-quality protein of aquaculture products, aquaculture has been the fastest-growing sector in food production over the past few decades [2]. However, the availability of freshwater resources is a significant limitation to aquaculture production [3]. It is important to explore alternative water sources, such as treated WWTP effluent, to meet the growing water demands of aquaculture. In general, WWTP effluent is rich in nutrients, which could support the growth of algae and plankton, providing natural feed for filter-feeding fish and reducing the feed costs [4,5]. Several studies have demonstrated the feasibility of using WWTP effluent for aquaculture [6,7,8]. For example, Zaibel et al. (2022) conducted a laboratory-scale experiment in which common carp were raised in tertiary-treated wastewater (TTWW) for five months, and no significant negative effects on fish survival, growth, or immune function were observed [3]. The levels of the hazardous substances in the fish tissue also remained below the safety standards. However, some other research has indicated that effluent can affect the survival rates of farmed aquatic species, particularly due to the emerging contaminants such as environmental hormones and antibiotics [9]. These pollutants have been demonstrated to have adverse effects on the reproductive system, resulting in developmental abnormalities [9].

According to the report by the World Health Organization (WHO), the use of steroid hormones in China is severe. In 2010, the annual emission of seven natural hormones already exceeded 3000 tons, significantly higher than that of the European Union and the United States [10]. For antibiotics, global consumption was approximately 200,000 tons in 2019. As one of the world’s largest producers and consumers, China’s per capita antibiotic usage increased by nearly 50% over the past decade [11]. These contaminants are introduced into WWTPs through domestic sewage, industrial wastewater, agricultural facility effluent, and aquaculture discharge. After a series of treatment processes such as sedimentation, anaerobic-anoxic-oxic (A^2^/O) process, and advanced purification (e.g., membrane filtration or advanced oxidation) in WWTPs, they are discharged into nearby water bodies. Previous studies have demonstrated that WWTPs could remove the environmental hormones and antibiotics through various treatment compartments [12,13]. However, it is noted that removal efficiencies varied due to different types of treatment, local legislation, and complex physicochemical properties of EOCs [14,15]. For example, sedimentation tanks are capable of removing certain organic contaminants, with removal efficiencies ranging from 13% (nonylphenol monoethoxylate) to 43% (bisphenol A, BPA) [16]. In contrast, primary treatment, such as aerated grit chambers, may lead to a notable increase in EOCs due to the stripping of compounds initially adsorbed onto grit during aeration [17]. Additionally, steroid estrogens, such as estrone and estradiol, are significantly degraded (>75%) during secondary treatment [18], whereas phenolic estrogens, like BPA, show lower biotransformation efficiency (56%) [18].

It is noted that the evaluation of removal efficiency of the organic contaminants (environmental hormones and antibiotics) in WWTPs is often based on instantaneous total concentrations, while the environmental impact of these organic contaminants is more closely related to their bioavailable concentrations (freely dissolved concentrations) [19]. Previous studies have indicated that the bioavailable concentrations of environmental hormones and antibiotics in WWTP influent and effluent were 2 to 10 orders of magnitude lower than their total concentrations [20,21]. The high particulate content in the influent adsorbs EOCs, thereby reducing their bioavailability [22]. Furthermore, high levels of dissolved organic carbon (DOC) could form complex compounds with metals and organic pollutants, altering their toxicity and bioavailable fractions [23,24]. This may lead to significant differences between the removal rates calculated based on total concentrations and those based on bioavailable concentrations. Passive sampling techniques serve as important tools for assessing bioavailable concentrations, including equilibrium-based samplers (e.g., PDMS) [25] and kinetic-based devices (e.g., ChemCatcher [26] and DGT [27]). Among these passive sampling tools, DGT samplers exhibited better specificity for ionizable antibiotics and environmental hormones [28,29,30]. In WWTP, Liang et al. [31] applied DGT samplers and found that quinolone (QNs) and tetracyclines (TCs) were of up to 1202.3 ng/L in the WWTP influent, and 830.1–966.3 ng/L in the effluent, indicating a warned removal efficiencies based on bioavailable concentrations. In natural waterbodies, DGT samplers have been applied widely. After a 14-day deployment, environmental hormones were found ranging from 0.89 to 5.1 ng/L in the UK [29], while varying from 2.2 to 3.6 ng/L in the creeks in France [32]. Previous studies have also simultaneously demonstrated that DGT provides a good prediction of antibiotics in organisms, further validating its capability to assess the bioavailable concentrations in the environment [33]. Therefore, it is more direct and effective to assess the removal of environmental hormones and antibiotics in WWTPs and their impact on aquatic organisms based on DGT samplers.

This study focuses on the environmental behaviors of EOCs through different processes in a typical WWTP in the megacity Shanghai and the environmental risk of the effluent and the receiving water body. By integrating DGT passive samplers and bioavailable concentration analysis, the objectives include: (1) investigating the environmental behavior patterns of environmental hormones and antibiotics along the WWTP based on DGT-measured bioavailable concentrations, and analyzing the removal efficiencies of target organic contaminants across different treatment units (influent, primary treatment, secondary biological treatment, and tertiary treatment), (2) assessing seasonal variations in the bioavailable concentrations of EOCs and identifying key factors influencing their removal during wastewater treatment, (3) identifying critical pollutants in the effluent and evaluating their potential for aquaculture reuse based on bioavailable concentrations and toxicity data for different aquatic organisms using the risk quotient (*RQ*) method, (4) determining the bioavailable concentrations of the target contaminants in the receiving water bodies affected by the WWTP effluent and assessing their potential as aquaculture zones. Overall, this study provides data support and a scientific basis for more accurate assessment and risk control of the studied analytes in and from WWTPs, as well as for evaluating the potential for aquaculture reuse of the effluent and the receiving natural water body.

## 2. Materials and Methods

### 2.1. Equipment and Reagents

The instruments used in the experiment included XAD-DGT devices (Nanjing Weisen Environmental Technology Co., Ltd., Nanjing, China), nitrogen evaporator (model QYN100-2, Shanghai Qiaoyue Electronic Co., Ltd., Shanghai, China), sonication apparatus (model KQ3200DB, Kunshan Ultrasonic Instrument Co., Ltd., Kunshan, China), liquid chromatography-triple quadrupole mass spectrometer (Agilent 1290 UPLC + 6460 MS/MS, Santa Clara, CA, USA), ultra-pure water system (Milli-Q^®^ Express 40, Merck Chemical Technology (Shanghai) Co., Ltd., Shanghai, China), medical low-temperature storage freezer (model DW-857, Chengdu Yike Medical Equipment Co., Ltd., Chengdu, China), solid-phase extraction apparatus.

The methanol, acetone, n-hexane, formic acid, acetonitrile, and formic acid used in the experiment were all of chromatographic grade and purchased from Shanghai Adamas Reagent Co., Ltd. (Shanghai, China) Disodium ethylenediaminetetraacetate (EDTA) and sodium chloride were of analytical grade and provided by Sinopharm Chemical Reagent Co., Ltd. (Shanghai, China). Sulfuric acid, hydrochloric acid, and ammonia water were of superior grade and purchased from Shanghai Kermel Reagent Co., Ltd. (Shanghai, China). The OASIS HLB cartridges (6 mL, 200 mg) were obtained from Varian (Lake Forest, CA, USA). The 0.45 μm glass fiber filter membranes (GF/F) and high-purity nitrogen gas (99.99%) were purchased from Whatman, UK, and Shanghai Lidan Industrial Gases Co., Ltd. (Shanghai, China), respectively. The target analytes and internal surrogates, including the sulfonamides group (SAs), like sulfadiazine (SD), sulfapyridine (SP), sulfamerazine (SMR), sulfamethoxazole (SMX), sulfadimethoxine (SDM); the fluoroquinolones group (FQs), including offoxacin (OFL), norffoxacin (NFX), ciproffoxacin (CFX), enroffoxacin (EFX); the tetracyclines (TCs) group, such as tetracycline hydrochloride (TC), oxytetracycline hydrochloride (OTC), chlortetracycline hydrochloride (CTC); the chloram phenicols group (CPs), including thiamphenicol (TP), florfenicol (FF), chloramphenicol (CP); the macrolides group (MLs), including erythromycin (EM), clarithromycin (CTM), roxithromycin (ROM); environmental hormones, bisphenol A (BPA), nonylphenol (NP), 17β-estradiol(E2), estriol (E3), as well as isotope-labeled substances such as SMX-*d*_4_, OFL-*d*_3_ CP-*d*_5_, EM-^13^C-*d*_3_, and BPA-*d*_14_, were purchased from Dr. Ehrenstorfer GmbH (Augsburg, Germany).

### 2.2. Sampling Sites in Different Compartments of the WWTP and Its Receiving Water Body

**Figure 1:**
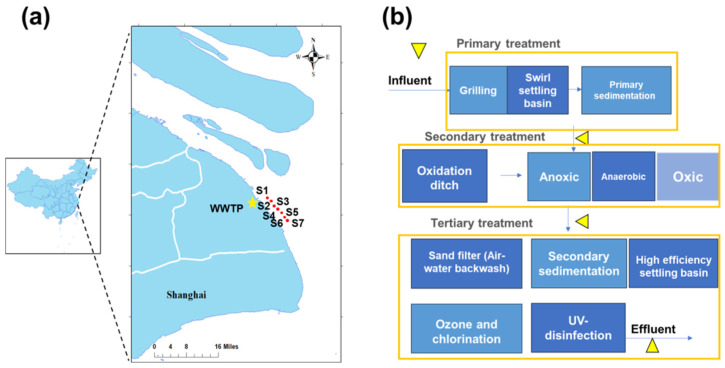
Location of the studied WWTP in the megacity Shanghai (**a**) and the major treatment components of the WWTP (**b**). The red dots in (**a**) and the yellow triangles in (**b**) are the sampling sites in the receiving water body and WWTP, respectively.

The target WWTP is located in Shanghai near the Yangtze River Estuary, with a capacity of 400,000 m^3^/d. It primarily receives domestic sewage, industrial wastewater, as well as some wastewater from livestock farms. The treated effluent is discharged into the Yangtze River Estuary via a submerged pipeline. As given in Figure 1, the primary treatment units include screens, grit chambers, and primary sedimentation tanks. The secondary treatment consists of an A^2^/O process followed by secondary sedimentation tanks. Tertiary treatment comprises sand filtration, ozone oxidation, and ultraviolet (UV) disinfection to achieve more efficient removal of pollutants. Meanwhile, samples from the water body that receives WWTP effluent were collected to assess the direct environmental impact. Seven sampling sites (S1–S7) were selected along the Yangtze River Estuary (Figure 1 and Table S1).

### 2.3. DGT Devices Application

XAD-DGT passive devices (n = 3) were placed simultaneously at the inlet and outlet of each treatment unit (influent, primary treatment, secondary treatment, and tertiary treatment) during both the dry season (March 2023) and the wet season (July 2023) using fixed installations (Figure 1). The devices should be used in areas with low turbulence, especially in low-bubble zones. During deployment, the DGT membrane should be kept fully submerged in wastewater for a period of 7 days. The water temperature was monitored throughout the deployment period. If the variation in water temperature was within 2 °C, the average value was used as the representative temperature for the deployment period. Upon the completion of passive sampling, the devices were retrieved from the water, and the DGT membrane, as well as the device housing, were gently rinsed with MiliQ water. The XAD-DGT devices were placed in aluminum foil bags, transported back to the laboratory, and stored at −20 °C before extraction.

The wastewater samples (V = 1 L and n = 3) were collected simultaneously and processed using the traditional pretreatment method. The details were provided in our previous studies [34]. Field water samples were then collected from the sampling sites in the receiving water body in the Yangtze River Estuary (Figure 1) (V = 1 L, n = 3) and stored in amber glass bottles, which were rinsed with MiliQ water and dried before use. The XAD-DGT devices (n = 3) were placed into the amber bottles for 7 days. The time point of deploying the devices and the corresponding water temperature were recorded. The XAD-DGT devices were rinsed and placed into the aluminum foil bags before extraction. In the meantime, water samples (V = 1 L, n = 3) for traditional sampling and extraction method were collected and processed according to our previous investigations [34].

### 2.4. Sample Extraction and Analytical Analysis

The filters and diffusion membranes of the DGT devices were removed using clean tweezers. Then, the binding membrane was carefully retrieved from the DGT sampler and placed into a 15 mL amber glass vial. HPLC-grade methanol was added (5 mL) for the sonication extraction (20 min) twice. The extracts were evaporated using nitrogen gas to nearly dryness and then re-dissolved in 1 mL of a 3:7 (*v*/*v*) acetonitrile/water solution. The final extract was stored at −18 °C until analysis by liquid chromatography-triple quadrupole mass spectrometry (Agilent 1290 UPLC + 6460 MS/MS, Santa Clara, CA, USA).

For the traditional sampling method, 1 L of water (filtered through 0.45 μm glass fiber filters) was extracted using Oasis HLB cartridges (500 mg, Waters, Milford, MA, USA). Before use, the cartridges were conditioned with 10 mL of methanol and 10 mL Milli-Q water and then loaded with water samples at a flow rate of 10 mL/min. The target contaminants retained on the cartridges were eluted with 8 mL methanol and 8 mL methanol: acetone (1:1, *v*/*v*). Finally, the eluates were evaporated to almost dry using a nitrogen evaporator and re-dissolved in 300 μL acetonitrile: water (3:7, *v*/*v*). The samples were transferred to a 1 mL vial and stored at −18 °C until UHPLC-MS/MS analysis.

The chemical analytical procedures are provided in our previously published study. In brief, the Agilent Zorbax RR Eclipse Plus C18 column (Santa Clara, CA, USA) (95 Å pore size, 3.5 μm particle size, 2.1 mm inner diameter, and 150 mm length) was applied, and the column temperature was 40 °C. The eluent A and eluent B were 0.1% formic acid/H_2_O and 0.05% methanoic acid/methanol at a flow rate of 0.3 mL/min for antibiotics. In the meantime, environmental hormones were analyzed using 0.1% ammonium hydroxide/H_2_O as eluent A and acetonitrile as eluent B at a flow rate of 0.3 mL/min. The injection volumes were 5 μL and 10 μL, respectively. For the ion source parameters in the mass spectrometry sector, nitrogen gas was used as drying and collision gas. The gas temperature was 300 °C, and the gas flow rates were 7 L/min (antibiotics) and 10 L/min (EDCs), respectively. The sheath gas temperature was 350 °C, the sheath gas flow rate was 11 L/ min, and the capillary was 3500 V. More specific instrument parameters, target compounds, multiple reaction monitoring (MRM) conditions, and the QA/QC data (Recovery and MDLs) for the analytes and internal standards are provided in Tables S2–S5.

### 2.5. Data Analysis

#### 2.5.1. DGT-Based Concentration Calculation

The DGT technique is developed based on Fick’s first law. The binding gel immediately adsorbs target analytes passing through the diffusion layer. The flux of a solute through a diffusion layer is proportional to the concentration gradient across it. Based on this fact, the DGT-measured concentration, denoted as *C_DGT_*, is then calculated using the following equation.

(1) $$C_{\mathrm{DGT}}=\frac{M(\Delta g+\delta )}{DAt}$$

where *M* is the mass of the target chemical accumulated on the binding gel, Δ*g* is the thickness of the diffusion membrane (0.08 cm), δs represents the thickness of the diffusion boundary layer (DBL). Generally, *δ* is significantly lower than Δ*g* and could be omitted in the calculation. *D* refers to the diffusion coefficient of the target analytes in the diffusion gel (Table S6), *A* refers to the sampling area of the DGT devices (3.14 cm^2^), and *t* is the deployment time.

#### 2.5.2. DGT-Derived Risk Assessment

The risk quotient is determined to quantify the environmental risk of the target analytes based on DGT-derived concentration. The calculations are as follows:

(2) $$RQ=\frac{MEC}{PNEC}$$

where *MEC* is the bioavailable concentration of individual environmental hormones and antibiotics measured in each sample, *PNEC* is the predicted no-effect concentration that is derived by dividing the 50% effect concentration (EC_50_) by an assessment factor (AF) [35,36,37]. AF values are usually defined as 1000 for acute toxicity and 100 for chronic toxicity. The *PNEC* values were calculated based on previous studies [34,38,39,40,41,42], and are given in Table S7. There is a high potential risk when *RQ* value ≥ 1, an intermediate risk when *RQ* value falls between 0.1 and 1, a low risk or negligible risk when *RQ* value < 0.1 [43,44]. At low concentrations of the target chemicals observed, the total risk (Σ*RQs*) was calculated by summing individual risk quotients based on the concentration addition (CA) model, while potential synergistic or antagonistic interactions were ignored [45].

## 3. Results and Discussion

### 3.1. Applicability of DGT in the Wastewater and Its Receiving Water Body

**Figure 2:**
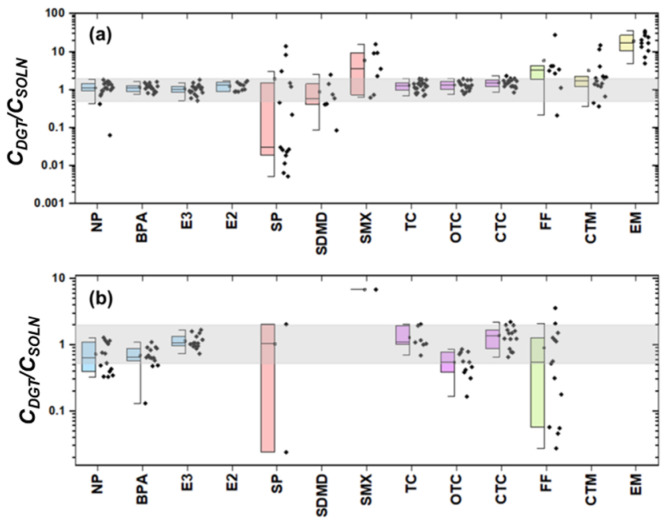
*C_DGT_*/*C_SOLN_* values of EDCs and antibiotics in the WWTP (**a**) and the effluent receiving water body (**b**). Note: the blue columns correspond to environmental hormones, the light red to SAs, the purple to TCs, the green to CPs and the yellow to MLs. The black dot represent the *C_DGT_*/*C_SOLN_* ratio in each sample. The results of other target analytes were not plotted in the figure, since these compounds were not detected using the XAD-DGT method.

The feasibility of using XAD-DGT for monitoring the bioavailable concentration of organic compounds in the WWTP and the receiving water was evaluated by the ratio of DGT measured and traditionally measured concentration in dissolved phase (*C_DGT_*/*C_SOLN_*). A ratio within the range of 0.50–2.0 was considered theoretically acceptable [46]. As shown in Figure 2a,b, only environmental hormones and antibiotics TCs could be measured by DGT device. Specifically, 98.1% of the *C_DGT_*/*C_SOLN_* values were observed within the acceptable range in WWTP, while 81.3% in the receiving water body. For other compounds, including SAs, CPs, and MLs, the *C_DGT_*/*C_SOLN_* ratios exhibited significant deviations from the reference range of 0.5–2.0. For FQs, no individual was detected in the DGT membrane.

Particularly in the WWTP, the median *C_DGT_*/*C_SOLN_* values for environmental hormones (NP, BPA, E3, and E2) were close to 1.0 (Figure 2a), indicating that the DGT is more effective at predicting these pollutants. This is primarily due to their moderate hydrophobicity (log K_OW_ = 2.45–4.77), leading to weak binding to particulate matter or dissolved organic carbon (DOC) in aquatic environments. Meanwhile, the high concentrations of the target contaminants make the fraction binding to DOC negligible [47]. For antibiotic compounds such as TC, OTC, and CTC, the *C_DGT_*/*C_SOLN_* values of 63.4% of the samples exceeded 1.5. Antibiotic compounds typically contain multiple functional groups (e.g., phenolic hydroxyl, enol, amide groups), which readily bind to metal ions [48] or DOC [49] in water. These complexes might be retained on the filters during filtration, leading to underestimated concentrations measured by the conventional sampling and extraction method [50,51]. In the meantime, the DGT samplers measured a 7-day average (TWA) concentration [30], and more pollutants might be adsorbed onto the binding gel.

It is noted that the performance of DGT in the natural water body was inferior (81.3%) to that in the WWTP (98.1%). As shown in Figure 2b, the DGT devices measured lower concentrations than the traditional method. The receiving water bodies undergo significant dilution, resulting in substantially lower pollutant concentrations. The DOC-bound fraction contributed a considerable proportion of the total concentration, leading to a significant reduction in the bioavailable concentration. It is noted that *C_DGT_*/*C_SOLN_* value of E3 was determined to be 1.1, suggesting a good prediction for E3. Generally, the compound E3 with intermediate hydrophobicity remains neutral in natural waters, leading to a limited binding to DOC. Then, the DGT devices overestimated TC and CTC concentration. The binding gel of the DGT devices is negatively charged, exhibiting strong adsorption for highly polar compounds (TC and CTC) that are positively charged in natural aquatic environments. Additionally, E2 was not detected by XAD-DGT in the receiving water bodies, which is likely due to its low ambient concentration in these environments.

In summary, the XAD-DGT devices were applicable for monitoring the bioavailable concentrations of environmental estrogens and antibiotics TCs in both WWTP and their receiving water bodies. However, due to varied environmental parameters and physicochemical properties of target pollutants, the reliability of XAD-DGT requires further optimization for various contaminants under different environmental conditions. In the following sections regarding the removal efficiencies and aquaculture reuse potentials, only environmental hormones and TCs with acceptable DGT recoveries were assessed.

### 3.2. Assessment of the Environmental Hormones and Antibiotics Removal in WWTP Based on the XAD-DGT Technique

#### 3.2.1. Environmental Hormones and Antibiotics in the Influent in WWTP

**Figure 3:**
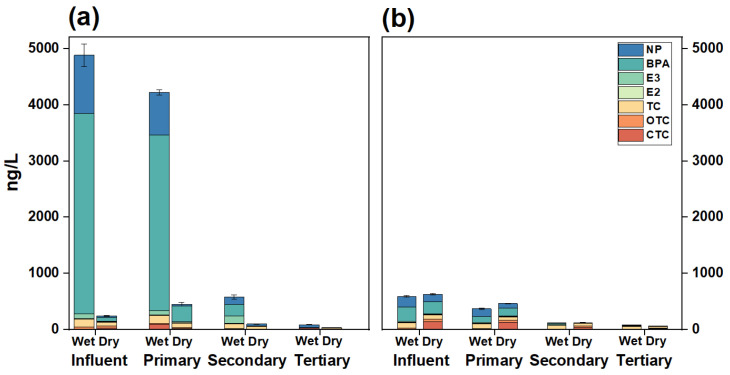
Environmental behavior of the seven environmental hormones and antibiotics in the WWTP based on DGT (**a**), traditional sampling method (**b**).

The behavior through different compartments and bioavailable concentrations of environmental hormones (NP, BPA, E3, and E2) and antibiotics (TC, OTC, CTC) measured by XAD-DGT in the WWTP are given in Figure 3a and Table 1. All seven contaminants were detected across different treatment processes, especially in the influent.

The total concentrations of environmental hormones in the influent were 4693.92 ± 203.31 ng/L during the wet season and 114.43 ± 1.19 ng/L during the dry season (Table 1). BPA and NP were identified as the primary contributors (Figure 3a). The concentrations of BPA were observed to be 3571.53 ± 198.19 ng/L and 68.90 ± 0.46 ng/L during the wet and dry seasons, respectively. In contrast, the concentration of NP was 1033.20 ± 41.44 ng/L in the wet season and 23.84 ± 0.085 ng/L in the dry season. The bioavailable concentrations of total antibiotics in the influent were 312.46 ± 12.67 ng/L and 129.86 ± 11.82 ng/L in the wet and dry seasons (Table 1). For each antibiotic individual, TC, OTC, and CTC were 144.44 ± 0.001 ng/L, 33.90 ± 0.11 ng/L, and 134.12 ± 12.67 ng/L in the wet season, whereas 64.26 ± 10.5 ng/L, 34.08 ± 0.55 ng/L and 31.52 ± 5.39 ng/L in the dry season (Table 1). The observed antibiotic concentrations in the present study were generally lower than those observed using DGT tools in North China (75.4 to 1202.3 ng/L) [31], indicating a difference in antibiotic use in these two areas.

It is noteworthy that the bioavailable concentrations of environmental hormones and antibiotics (e.g., TC and CTC) measured by DGT were significantly higher during the wet season than during the dry season. Wastewater discharge from livestock farming introduces a high load of antibiotics into the WWTPs [52,53]. In the wet season, livestock production activities are more extensive [52,53], leading to elevated concentrations in the influent of WWTPs [54]. In addition, high antibiotic load in the wet season can be primarily related to the elevated temperatures during the wet season (July), which strengthens the activity of microbial hydrolytic enzymes, thereby promoting the hydrolysis of contaminants and leading to an increase in their freely dissolved concentrations (i.e., bioavailable fraction) [55]. The high temperature not only accelerates hydrolysis but also enhances microbial metabolic activity in wastewater treatment systems [56,57]. This promotes the release of contaminants adsorbed onto the particulate matter, thereby increasing the dissolved proportion of the pollutants in the wastewater, which are more readily captured by DGT. Compared to the concentrations measured by conventional methods (Figure 3b), the values obtained via DGT were significantly higher, particularly during the wet season. This discrepancy may be due to the fact that DGT sampling measures TWA concentration, whereas conventional sampling reflects instantaneous concentrations.

#### 3.2.2. XAD-DGT Derived Removal Efficiency

In the effluent, the DGT-derived concentrations were reduced. The total concentrations of environmental hormones in the effluent were 60.14 ± 2.76 ng/L during the wet season and 2.78 ± 0.14 ng/L during the dry season (Table 1). The major contributors were BPA and NP as well. The DGT measured antibiotics were 26.8 (major contributor CTC) during the wet season, while 32.36 ± 1.40 (major contributor TC) during the dry season (Table 1).

**Figure 4:**
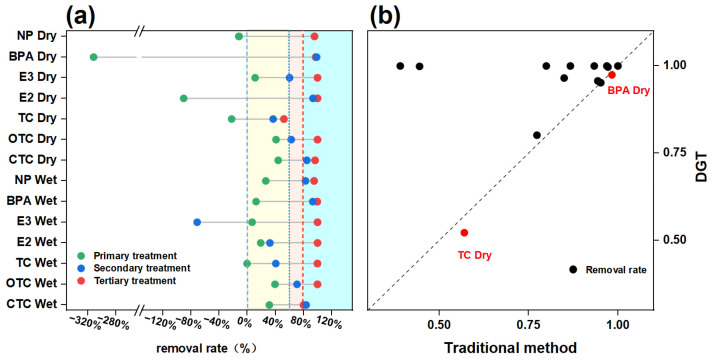
Removal rate of each treatment unit (**a**) and a comparison between the removal rates based on DGT and the traditional sampling method (**b**). Note: The red dots in figure (**b**) identify BPA and TC as compounds for which the traditional method measured a lower removal rate.

The removal performance of the contaminants studied in terms of bioavailable concentrations varied significantly across primary, secondary, and tertiary processes under different seasons (Figure 4a). During the wet season, removal efficiencies of primary, secondary, and tertiary processes for the environmental hormones were 15.53%, 88.03%, and 87.32%, respectively, and secondary and tertiary processes performed better. For antibiotics, the primary and secondary processes exhibited limited removal efficiency, with elimination rates of 16.73% and 7.92%, respectively. However, the tertiary process achieved significantly higher removal, with an efficiency of 92.73%. During the dry season, the treatment processes exhibited different trends from those observed in the wet season. The DGT-measured concentrations of environmental hormones increased by 79.02% and 193.91%, respectively, in the primary treatment process. The secondary process exhibited limited performance in removing antibiotics but demonstrated high removal efficiency (93.62%) for environmental hormones. Similarly, the tertiary treatment achieved effective removal of both types of contaminants. In summary, during the wet season, although the influent loading surged, environmental hormones were effectively removed in the secondary process due to their hydrophobicity through adsorption [58]. In contrast, the removal of antibiotics was depressed by the reduced HRT and SRT, resulting from high hydraulic loading rates, which inhibited the activity of specialized degradation microorganisms [59]. In the dry season, an increase in environmental hormones and antibiotics was observed in the primary compartment. This is primarily due to the extended HRT, enhancing the bio-hydrolysis and desorption of particle-bound contaminants in the sedimentation tank, thereby releasing freely dissolved (bioavailable) fractions into the aqueous phase [60]. The tertiary treatment process performed stately and efficiently, and it could be considered a critical tool for controlling the emerging contaminants, including environmental hormones and antibiotics.

Substantial differences were observed in the removal efficiencies of bioavailable concentrations for different individuals (Figure 4a and Table S8). The primary treatment process exhibited limited removal efficiency for all target individuals across both seasons. The sedimentation tanks in the primary processes are designed to remove larger particulate matter and are generally ineffective at eliminating dissolved substances. For individuals such as TC, NP, and BPA, a release was observed during the dry season. This may be attributed to the prolonged HRT, which promoted the desorption and hydrolysis of particle-bound contaminants in the sedimentation tank. The secondary treatment process performed well, and the removal rates were as high as 84.75%, 82.84%, 98.75% and 93.62% for CTC, NP, BPA, and E2, whereas it performed unacceptable removal rates for TC and E3, ranging from −71.35 to 60.27%. Firstly, the influent concentrations were low for TC and E3, leading to insignificant removal [61]. Then, the high polarity of E3 limits its partitioning into hydrophobic sludge flocs, thereby reducing its bioaccessibility and restraining the removal efficiency [62]. Finally, the tertiary treatment performed well (>80%) for all the target organic chemicals (Figure 4a, Table S8). This further confirmed that tertiary treatment (high-efficiency sedimentation tanks, sand filters, ozone, chlorination units, and disinfection), which primarily relies on adsorption and advanced oxidation processes, could effectively reduce the bioavailable fraction of contaminants in wastewater. Consequently, they significantly mitigated the direct impact of effluent-derived target contaminants on the aquatic organisms in the receiving water bodies. Also, as suggested by Figure 4b, the removal rates derived from XAD-DGT measurements were comparable to or exceeded those obtained using conventional methods. This indicated that the wastewater treatment processes were more effective at reducing the bioavailable fraction (i.e., freely dissolved concentration), rather than the total concentration.

In summary, the higher removal efficiency observed for the bioavailable fraction highlighted the importance of adopting bioavailability-based assessments for evaluating the actual ecological risk reduction achieved by the WWTP.

### 3.3. Feasibility of Using WWTP Effluent and Receiving Water for Aquaculture: From Bioavailable Perspectives

The XAD-DGT derived ecological risks posed by environmental hormones and antibiotics to fish, crustaceans, and algae were significantly reduced through WWTP. In the wet season, the risk to fish decreased by a range of 99.98% through the WWTP (Table S9), posing a low risk with an effluent Σ*RQ* value of 0.0051, primarily attributable to NP. In the dry season, the risk reduction was 99.89% (Table S9), and the remaining environmental hormones and antibiotics also presented a low risk to fish, with an effluent Σ*RQ* value of 0.044. For crustaceans, the environmental risks decreased by a rate of 74.68% during the wet season and 95.45% during the dry season. The effluent *RQ* values were 0.020 (low risk) and 0.15 (moderate risk), primarily due to environmental hormones, NP, and E2. For algae, the residual environmental hormones and antibiotics in the WWTP effluent posed a low ecological risk during both the wet and dry seasons, with effluent *RQ* values of 0.019 (low risk) and 0.12 (moderate risk), respectively. The risk driven for algae was environmental hormones (NP) in general. In summary, the bioavailable concentrations of environmental hormones and antibiotics in WWTP effluent posed low ecological risks to fish. For crustaceans and algae, NP may pose a moderate ecological risk. From this perspective, the effluent water is applicable for fish aquaculture reuse; however, further removal techniques for effluent water, like adsorption or advanced oxidation, should be applied before the reuse for crustaceans, especially for contaminants like environmental hormones. Additionally, some studies suggested that an *RQ* < 1 is considered a low risk [63]. According to this criterion, the treated effluent showed a potential for direct reuse in both fish and crustacean aquaculture.

**Figure 5:**
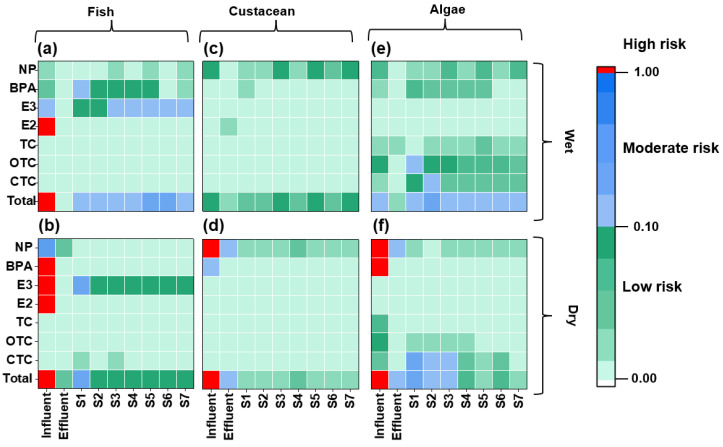
Environmental risk assessment (*RQs*) of the target contaminants for fish (**a**,**b**), crustacean (**c**,**d**), and algae (**e**,**f**) in the influent, effluent, and receiving waterbody. The upper panels (**a**,**c**,**e**) showed *RQs* values during the wet season, while the lower panels (**b**,**d**,**f**) showed those during the dry season.

The environmental risk posed by the environmental hormones and antibiotics in the receiving water bodies of the WWTP using XAD-DGT was evaluated to indicate the direct environmental impact of the effluent from the WWTP more accurately (Figure 5 and Table S9). Notably, the Σ*RQ* of the studied contaminants in the receiving water bodies for fish ranged from 0.085 to 0.32. The overall risk was at a moderate level in the wet season, primarily driven by the combined effects of E3 and BPA, whereas the majority of the sites exhibited low risk in the dry season, except one site S1 (moderate risk triggered by E3). In the meantime, the ecological risk posed by the target contaminants in the receiving water bodies was lower for crustaceans than for fish. Specifically, the Σ*RQ* values ranged from 0.032 to 0.089 during the wet season and from 0.016 to 0.032 during the dry season (Figure 5 and Table S9), with NP identified as the primary risk contributor. For algae, the Σ*RQ* values varied between 0.09 and 6.8. During the wet season, all the sites exhibited moderate risk levels, ranging from 0.15 to 0.32. The antibiotics OTC and CTC were the major risk contributors during this period. During the dry season, half of the sampling sites presented moderate risks, while the remaining sites showed low risk. The compound CTC was the primary driver. Comparatively, the studied area exhibited lower risk values (CTC, *RQ* = 0.1, Table S9), than those in an investigation based on DGT-derived risk assessment in South China (CTC, *RQ* = 0.1–0.28) [64]. Seasonal variation regarding environmental risk was observed. The ecological risks of the receiving water to fish, crustaceans, and algae in the wet season were at low to moderate risk levels, and consistently higher than those during the dry season. There are several WWTPs upstream, and the risk values ranged from 0.28 to 30.4 for fish and 1.72 to 4.54 for algae [34] during the wet season. The upstream effluent and river water might impact the environmental risk more significantly than the studied WWTP effluent. Since aquaculture activities are generally scheduled in the wet season, the areas in proximity to the WWTP effluent outlet are only feasible for crustacean aquaculture (Figure 5). Also, the environmental risk in the receiving water body was surprisingly higher than that of the WWTP effluent at some sampling sites, further confirming a higher environmental impact of contaminants from the upstream water body.

Therefore, the effluent water was applicable for fish aquaculture reuse, but further removal techniques for environmental hormones should be applied before the reuse for crustaceans. The receiving water bodies were not feasible for fish aquaculture, but were applicable for crustaceans. While the ecological risk of the WWTP effluent was comparable or lower than that of the downstream water, its viability as a reclaimed water resource was underestimated.

## 4. Conclusions

This study employed DGT passive sampling tool to investigate the bioavailable fractions of environmental hormones and antibiotics, which is a more biologically and ecologically relevant metric, in a typical Shanghai wastewater treatment plant (WWTP) and its relevant water bodies. It is more novel to evaluate the reuse potential of effluent and the relevant water body for aquaculture based on the DGT samplers. Key findings indicated that DGT effectively captured the bioavailable pollutants within the complex WWTP matrix compared to the receiving water environment. Removal efficiencies exhibited significant seasonal and technological variations. The tertiary treatment efficiently eliminated both environmental hormones and antibiotics (>80%). The primary treatment elevated the target pollutant concentrations during the dry season due to desorption, while secondary treatment showed a variability in removal performance depending on the physicochemical properties of the contaminants. The effluent posed low ecological risks to fish but moderate ecological risks for crustaceans and algae. Thus, the effluent water is applicable for fish aquaculture reuse; however, further removal techniques for environmental hormones should be applied before the aquaculture reuse for crustaceans. The receiving water bodies posed low to moderate risk to fish, driven by BPA and E3; low risk to crustaceans, mainly induced by NP; and low to moderate risk to algae, with antibiotics being the major risk drivers. Environmental remediations should be conducted in these areas before they are applied for aquaculture. More importantly, WWTP effluent presents a lower ecological risk than the downstream receiving water, highlighting its underestimated potential as a managed water resource. Finally, in the present investigation, DGT was only feasible for a limited range of chemicals. The application of DGT across heterogeneous environmental media for more chemicals warrants future study.

## Figures and Tables

**Table 1:** The residual levels of the environmental hormones and antibiotics in different treatment compartments in the selected WWTP in Shanghai.

Chemicals	Influent	Primary Treatment Effluent	Secondary Treatment Effluent	Tertiary Treatment Effluent
	Wet Season			
BPA	3571.53 ± 198.19	3122.52 ± 43.43	210.75 ± 33.39	10.69 ± 0.69
NP	1033.2 ± 41.44	760.42 ± 22.92	130.53 ± 2.57	49.45 ± 2.67
E2	8.66 ± 0.05	7.01 ± 0.08	4.75 ± 0.61	/
E3	80.53 ± 4.13	74.95 ± 4.55	128.43 ± 1.16	/
∑EDCs	4693.92 ± 202.52	3964.9 ± 49.32	474.45 ± 33.512	60.14 ± 2.76
TC	144.44	144.85 ± 1.7	86.03 ± 2.44	0.17
OTC	33.9 ± 0.11	20.53 ± 0.25	5.99 ± 0.11	/
CTC	134.12 ± 12.67	92.05 ± 5.18	14.91 ± 1.44	26.63
∑Antibiotics	312.46 ± 12.67	257.43 ± 5.46	106.93 ± 2.84	26.8
∑EOCs	5006.38 ± 203.31	4365.43 ± 49.92	843.26 ± 33.75	86.94 ± 2.76
	**Dry Season**			
BPA	68.9 ± 0.46	283.59 ± 5.93	3.54 ± 0.06	1.76 ± 0.08
NP	23.84 ± 0.09	26.58 ± 0.01	29.78 ± 2.73	1.02 ± 0.12
E2	6.78 ± 0.77	12.92 ± 1.28	0.82 ± 0.12	/
E3	14.91 ± 0.78	13.23 ± 0.84	5.26 ± 0.42	0.2
∑EDCs	114.43 ± 1.19	336.32 ± 6.12	39.39 ± 2.77	2.78 ± 0.14
TC	64.26 ± 10.5	78.58 ± 16.35	49.68 ± 3.27	30.7 ± 0.77
OTC	34.08 ± 0.55	0.83 ± 0.83	0.83 ± 0.83	0.83 ± 0.83
CTC	31.52 ± 5.39	0.83 ± 0.83	0.83 ± 0.83	0.83 ± 0.83
∑Antibiotics	129.86 ± 11.82	80.24 ± 16.40	51.34 ± 3.47	32.36 ± 1.40
∑EOCs	244.29 ± 16.75	416.56 ± 17.50	843.26 ± 5.64	86.94 ± 1.99

## Data Availability

The original contributions presented in this study are included in the article/Supplementary Materials. Further inquiries can be directed to the corresponding author.

## Supplementary Materials

The following supporting information can be downloaded at: https://www.mdpi.com/article/10.3390/toxics13110970/s1, Table S1. Information of the sampling locations. Table S2. Instrumental parameters for the target compounds. Table S3. Mass spectrum monitoring conditions of target compounds and internal standards. Table S4. Physicochemical properties of the target contaminants. Table S5. The recoveries (%), method detection limits (MDLs), and limits of quantification (LOQs) of the contaminants. Table S6. Diffusion coefficient D of target analytes in diffusion gel. Table S7. PNECs values used in environmental risk assessment. Table S8. Removal efficiencies of environmental hormones and antibiotics of each treatment compartment. Table S9. Environmental risk assessment of environmental hormones and antibiotics of the influent, effluent compartment, and the receiving water body.
