# Supplementary material for: DGT-Based Assessment of Antibiotics and Hormones in a Typical Wastewater Treatment Plant and Its Receiving Water in Shanghai: Implications for Aquaculture Reuse
Source: Toxics. 2025 Nov 11;13(11):970. doi: 10.3390/toxics13110970 (PMC12656330; doi:10.3390/toxics13110970)
Supplement: Supplementary file 1 [file toxics-13-00970-s001.zip › toxics-3912961-supplementary.pdf]

Supplementary Materials

DGT-Based Assessment of Antibiotics and Hormones in a Typical WWTP and Its Receiving Water in Shanghai: Implications for Aquaculture Reuse

Huang Yin [1,#], Zhang Zheng [2,#], Sun Chaofeng [3], Wen Luting [1], Wang Qian [2,*], Yang Yanhao [1]

[1] Guangxi Key Laboratory of Aquatic Genetic Breeding and Healthy Aquaculture, Guangxi Institute of Fisheries, Nanning, Guangxi, China
[2] College of Oceanography and Ecological Science, Shanghai Ocean University
[3] CTIesting International Group Co., Ltd

[*]Address correspondence to Wang Qian; Email: q_wang@shou.edu.cn; Tel: + 021 6190 0431; FAX: + 021 6190 0431
[#] The authors contributed to the article equally as first authors.

Table S1 Information of the sampling locations.

Sampling sites	Latitude and longitude
WWTP	121.8841N, 31.0721E
S1	121.9119N,31.0555E
S2	121.9170N,31.0464E
S3	121.9171N,31.0465E
S4	121.9364N,31.0165E
S5	121.9459N,30.9982E
S6	121.9523N,30.9825E
S7	121.9591N,30.9680E

Note: The WWTP collects mechanical processing wastewater, electronic manufacturing wastewater, chemical manufacturing wastewater, and textile dyeing wastewater.

Table S2 Instrumental parameters for the target compounds.

Antibiotics	Ionization mode		Positive/Negative ionization						
	LC conditions	Mobile phase	A	0.1% formic acid/H_2O					
			B	0.05% methanoic acid/methanol					
		Gradient table	Time (min)	0	0.5	2	6	10	10.1
			A（%）	80	80	60	5	5	80
			B（%）	20	20	40	95	95	20
		Total flow	0.3mL/min						
		Cartridge	Agilent Zorbax RR Eclipse Plus C18 column (95 Å pore size, 3.5 µm particle size, 2.1 mm inner diameter and 150 mm length).						
		Cartridge temperature	40℃						
	MS conditions	Gas temperature	300℃						
		Gas flow	7L/min						
		Sprayer	45psi						
		Sheath gas flow	11L/min						
		Sheath gas temperature	350℃						
		Capillary	3500V						
		Nozzle voltage	500V						
Environmental hormones	Ionization mode		Negative ionization						
	LC conditions	Mobile phase	A	0.1% ammonium hydroxide/H_2O					

		B	acetonitrile						
	Gradient table	Time (min)	0	0.5	2	12	13	13.1	15
		A（%）	100	50	50	0	0	100	100
		B（%）	0	50	50	100	100	0	0
	Total flow	0.3mL/min							
	Cartridge	Agilent Zorbax RR Eclipse Plus C18 column (95 Å pore size, 3.5 µm particle size, 2.1 mm inner diameter and 150 mm length).							
	Cartridge temperature	40℃							
MS conditions	Gas temperature	300℃							
	Gas flow	10L/min							
	Sprayer	45psi							
	Sheath gas flow	11L/min							
	Sheath gas temperature	350℃							
	Capillary	3500V							
	Nozzle voltage	500V							

Table S3 Mass spectrum monitoring conditions of target compounds and internal standards.

Contaminants	Precursor ion (m/z)	Productions (m/z)	Fragmentor voltage (V)
SD	251.1	65(56);92.1(32)	95
SP	250.1	65.1(56);92.1(32)	100
SMR	265	65.1(60);92.1(36)	90
SMX	254	65.1(56);92.1(28)	85
SDM	311.1	156(20);65(68)	115
FLE	370.2	269(28);326.2(20)	125
OFL	362.2	261.1(28);318.2(20)	115
NFX	320.2	302.1(20);231.1(44)	115
CFX	332.2	231(44);314.1(20)	115
EFX	360.2	342.2(20);245.1(28)	110
SFX	386.1	368.1(24);299.1(32)	45
TC	445.3	410(20);427.2(12)	135
OTC	461.2	426.3(20);443.3(12)	95
CTC	479.1	444(20);462.1(16)	130
DC	445.2	428.1(16);154(28)	95
TP	354	185.1(16);290.2(8)	131
FF	356	336(4);185(16)	116
CP	321	152(12);257.1(4)	114
EM	734.4	158.1(32);116(56)	145
CTM	748.4	158.1(32);116.1(52)	175

ROM	837.5	158.1(40);679.4(20)	160
BPA	227.1	212.1(16);133(28)	115
NP	219.3	132.9(36);147(28)	95
E2	271.2	145(40);183(44)	100
E3	287.2	144.9(40);170.9(32)	115

Table S4 Physicochemical properties of the target contaminants.

Category	Compound	Acronym	CAS No.	Formula	MW	pK_a	logK_{ow}	Internal Standard	Supplier
Sulfonamides (SAs)	Sulfadiazine	SD	68-35-9	$C_{10}H_{10}N_4O_2S$	250.30	1.57 ± 0.10<br>6.50 ± 0.30	–0.12		Dr. Ehrenstorfer GmbH (Augsburg, Germany)
Sulfonamides (SAs)	Sulfapyridine	SP	144-83-2	$C_{11}H_{11}N_3O_2S$	249.29	2.58/8.43/8.4	0.35		
Sulfonamides (SAs)	Sulfamerazine	SMR	127-79-7	$C_{11}H_{12}N_4O_2S$	264.30	2.82 ± 0.31<br>6.84 ± 0.30	0.34	SMX-d4	
Sulfonamides (SAs)	Sulfamethoxazole	SMX	723-46-6	$C_{10}H_{11}N_3O_3S$	253.28	1.85 ± 0.30<br>5.60 ± 0.04	0.89		
Sulfonamides (SAs)	Sulfadimethoxine	SDM	122-11-2	$C_{12}H_{14}N_4O_4S$	310.33	2.01 ± 0.30<br>7.11 ± 0.09	0.05		
Fluoroquinolones (FQs)	Fleroxacin	FLE	79660-72-3	$C_{17}H_{18}F_3N_3O_3$	369.34	-0.54/7.15	0.24	CFX-d8	
Fluoroquinolones (FQs)	Ofloxacin	OFL	82419-36-1	$C_{18}H_{20}FN_3O_4$	362.15	2.27 ± 0.40<br>6.41 ± 0.30	1.41		
Fluoroquinolones (FQs)	Ciprofloxacin	CFX	85721-33-1	$C_{17}H_{18}FN_3O_3$	331.34	3.01/6.14/8.7/10.5/8	0.4		
Fluoroquinolones (FQs)	Enrofloxacin	EFX	93106-60-6	$C_{19}H_{22}FN_3O_3$	359.39	3.85 ± 0.30<br>6.19 ± 0.18	1.1		
Fluoroquinolones (FQs)	Sarafloxacin	SFX	91296-87-6	$C_{20}H_{18}ClF_2N_3O_3$	421.83				

Category	Compound	Acronym	CAS No.	Formula	MW	pK_a	logK_{ow}	Internal Standard	Supplier
	hydrochloride								
Fluoroquinolones (FQs)	Norfloxacin	NFX	70458-96-7	$C_{16}H_{18}FN_3O_3$	319.33	3.11/6.10/8.6/10.5/6	-1.03		
Tetracyclines (TCs)	Tetracycline hydrochloride	TC	64-75-5	$C_{22}H_{24}N_2O_8 \cdot HCl$	480.9	3.3	-1.19	NFX-d5	
Tetracyclines (TCs)	Oxytetracycline hydrochloride	OTC	79-57-2	$C_{22}H_{24}N_2O_9$	460.43	3.27 ± 0.10<br>7.32 ± 0.04<br>9.11 ± 0.10	–1.5		
Tetracyclines (TCs)	Chlortetracycline hydrochloride	CTC	64-72-2	$C_{22}H_{23}ClN_2O_8$	478.9	3.30 ± 0.40<br>7.40 ± 0.13<br>9.27 ± 0.50	–0.33	NFX-d5	
Tetracyclines (TCs)	Doxycycline hyclate	DC	24390-14-5	$C_{22}H_{24}N_2O_8 \cdot HCl$	480.9	3.02/7.97/9.15	2.37		
Chloram Phenicols(CPs)	Thiamphenicol	TP	15318-45-3	$C_{12}H_{15}Cl_2NO_5S$	356.22	-2.8/7.65	-0.27		
Chloram Phenicols (CPs)	Florfenicol	FF	73231-34-2	$C_{12}H_{14}Cl_2FNO_4S$	358.21	9.0	0.37	CP-d5	
Chloram Phenicols (CPs)	Chloramphenicol	CP	56-75-7	$C_{11}H_{12}Cl_2N_2O_5$	323.13	5.5	1.14		
Macrolides (MLs)	Erythromycin	EM	114-07-8	$C_{37}H_{67}NO_{13}$	733.93	8.90 ± 0.15	2.83		
Macrolides (MLs)	Clarithromycin	CTM	81103-11-9	$C_{38}H_{69}NO_{13}$	747.95	7.25	3.16	EM-13C-d3	
Macrolides (MLs)	Roxithromycin	ROM	80214-83-1	$C_{41}H_{76}N_2O_{15}$	837.53	9.17 ± 0.30	3.73		
Phenolic estrogenic compounds (PEs)	Bisphenol A	BPA	80-5-7	$C_{15}H_{16}O_2$	228.3	10.2	3.81		
Phenolic estrogenic compounds (PEs)	Nonylphenol	NP	25154-52-3	$C_{15}H_{24}O$	220.3	9.8	5.66	BPA-d14	
Steroid estrogens (SEs)	17β-Estradiol	E2	50-28-2	$C_{18}H_{24}O_2$	272.4	10.3	3.57		

Category	Compound	Acronym	CAS No.	Formula	MW	pK_a	logK_{ow}	Internal Standard	Supplier
Steroid estrogens (SEs)	Estriol	E3	50-27-1	$C_{18}H_{24}O_3$	288.4	10.3	2.54		
Internal Standard	Sulfamethoxazole-d4	SMX-d4	1020719-86-1	$C_{10}H_{11}N_3O_3S$	257.30				
Internal Standard	Ciprofloxacin-d8	CFX-d8	1130050-35-9	$C_{17}H_{18}FN_3O_3$	339.39				
Internal Standard	Erythromycin-13C-d3	EM-13C-d3	959119-26-7	$C_{37}H_{67}D_3N_2O_{12}$	737.99				
Internal Standard	Norfloxacin-d5	NFX-d5	1015856-57-1	$C_{16}H_{13}D_5FN_3O_3$	324.36				
Internal Standard	Chloramphenicol-d5	CP-d5	202480-68-0	$C_{11}H_7D_5Cl_2N_2O_5$	328.16				
Internal Standard	Bisphenol A-d14	BPA-d14	120155-79-5	$C_{15}H_{16}O_2$-D_{14}	242.2				

Table S5 The recoveries (%), method detection limits (MDLs), and limits of quantification (LOQs) of the contaminants.

Contaminants	Internal Standard	R^2	Recovery (%)	MDLs（μg/L）
SD	SMX-d4	0.9996	97.3	0.0814
SP	SMX-d4	0.9999	98.1	0.0752
SMR	SMX-d4	0.9991	98.3	0.0848
SMX	SMX-d4	0.9997	95.8	0.0751
SDM	SMX-d4	0.9992	101	0.0537
FLE	CFX-d8	0.9993	105	0.452
OFL	CFX-d8	0.9992	102	0.452
NFX	NFX-d5	0.9997	99.7	0.0682
CFX	CFX-d8	0.9994	105	0.445
EFX	CFX-d8	0.9976	105	0.445
SFX	CFX-d8	0.9999	103	0.479
TC	NFX-d5	0.9975	106	1.65
OTC	NFX-d5	0.9994	109	0.465
CTC	NFX-d5	0.9993	104	1.27
DC	NFX-d5	0.9998	109	0.445
TP	CP-d5	0.9997	103	0.486
FF	CP-d5	0.9998	98.0	0.0893
CP	CP-d5	0.9998	100	0.0893
EM	EM-^{13}C-d_3	0.9999	89.7	0.0552
CTM	EM-^{13}C-d_3	0.9997	88.6	0.0634
ROM	EM-^{13}C-d_3	0.9997	86.9	0.0718
BPA	BPA-d14	0.9993	99.2	0.0746

NP	BPA-d14	0.9996	85.6	0.369
E2	BPA-d14	0.9988	88.5	0.476
E3	BPA-d14	0.9968	94.0	0.667

Table S6 Diffusion coefficient D of target analytes in diffusion gel.

Contaminants	D (10^{-6} cm^2/s)
BPA	4.80
NP	4.13
E2	3.58
E3	4.59
SDMD	4.10
SMX	5.00
SP	4.80
OFL	2.20
TC	1.34
OTC	1.25
CTC	1.91
FF	1.03
CTM	1.95
EM	1.85

Table S7 PNECs values used in environmental risk assessment.

Substances	Algae (ng/L)	Fish (ng/L)	Crustaceans (ng/L)
CTC	1900	8000	85000
OTC	170	1200	110000
TC	7890	72920	200000
NP	721	330	35000
BPA	1000	60	1500000
E2	2480	1	800
E3	208390	46.2	2500

Note: The toxicity data were collected from previous studies [1-6].

Table S8 Removal efficiencies of environmental hormones and antibiotics of each treatment compartment.

		Primary treatment	Secondary treatment	Tertiary treatment	Total
Wet	BPA	12.57%	93.25%	94.93%	99.70%
	NP	26.40%	82.84%	62.11%	95.21%
	E3	6.93%	-71.35%	100.00%	100.00%
	E2	19.13%	32.27%	100.00%	100.00%
	∑EDCs	15.53%	88.03%	87.32%	98.72%
	TC	-0.28%	40.61%	99.81%	99.89%
	OTC	39.42%	70.83%	100.00%	100.00%
	CTC	31.37%	83.81%	-78.67%	80.14%
	∑antibiotics	16.73%	7.92%	92.73%	94.43%
	∑EDCs+ ∑antibiotics	15.64%	80.68%	89.69%	98.32%
Dry	BPA	-311.59%	98.75%	50.13%	97.44%
	NP	-11.51%	-12.02%	96.57%	95.72%
	E3	11.25%	60.27%	96.19%	98.66%
	E2	-90.46%	93.62%	100.00%	100.00%
	∑EDCs	-193.91%	88.29%	92.93%	97.57%
	TC	-22.30%	36.78%	38.21%	52.22%
	OTC	40.90%	62.56%	100.00%	100.00%
	CTC	44.09%	84.75%	59.49%	96.54%
	∑antibiotics	-79.02%	43.55%	90.80%	90.70%
	∑EDCs+ ∑antibiotics	-107.83%	59.41%	91.02%	92.42%

Table S9 Environmental risk assessment of environmental hormones and antibiotics of the influent, effluent compartment, and the receiving water body.

Fish																		
	Wet									Dry								
	Influent	Effluent	S1	S2	S3	S4	S5	S6	S7	Influent	Effluent	S1	S2	S3	S4	S5	S6	S7
BPA	2.55109	0.00764	0.00199	0.00247	0.00203	0.00121	1.06E-03	9.29E-04	8.07E-04	0.04921	0.00126	0.1234	0.09956	0.08766	0.08398	0.07703	0.00881	0.01449
NP	0.6888	3.30E-02	3.90E-03	2.80E-03	4.08E-03	7.04E-03	0.00349	0.00557	0.00353	0.01589	6.80E-04	0.00672	0.00605	0.01715	0.00521	0.01819	0.00711	0.01911
E2	36.08333	0	0	0	0	0	0	0	0	28.25	0	0	0	0	0	0	0	0
E3	1.20194	0.00299	0.3106	0.09746	0.08448	0.08537	0.0794	0.07761	0.08493	0.22254	0.00299	0.07463	0.09746	0.12179	0.14761	0.21836	0.28179	0.25403
TC	5.54E-04	6.53E-07	0.00E+00	0.00E+00	0.00E+00	0.00E+00	0.00E+00	0.00E+00	0.00E+00	5.55E-04	1.18E-04	6.57E-05	8.58E-05	1.07E-04	1.41E-04	2.65E-04	2.12E-04	2.24E-04
OTC	1.03E-04	0.00E+00	1.51E-05	1.44E-05	2.28E-05	1.42E-05	0.00E+00	0.00E+00	0.00E+00	1.69E-04	2.52E-06	1.35E-04	9.97E-05	8.69E-05	6.07E-05	5.54E-05	5.62E-05	4.94E-05
CTC	2.19E-03	4.35E-04	0.01581	0.00719	0.01079	0.0016	5.52E-04	0.00196	3.25E-04	4.09E-02	1.36E-05	0.00412	0.00754	0.00202	0.00222	0.00167	0.00227	0.00213

∑EDCs+∑antibiotics	40.528007	40.528007	0.33	0.11	0.10	0.10	0.08	0.09	0.09	8.360	0.030	0.030	0.030	0.030	0.030	0.030	0.030	0.290
Crustacean																		
	Wet									Dry								
	Influent	Effluent	S1	S2	S3	S4	S5	S6	S7	Influent	Effluent	S1	S2	S3	S4	S5	S6	S7
BPA	0.23042	6.90E-04	2.23E-04	1.83E-04	1.09E-04	9.61E-05	8.39E-05	7.29E-05	23.21%	0.00445	1.14E-04	0.01115	0.00899	0.00792	0.76%	0.70%	7.96E-04	0.13%
NP	3.06588	0.14674	0.01246	1.82%	3.13%	1.55%	2.48%	1.57%	334.80%	0.07074	0.00303	0.02991	0.02694	0.07635	2.32%	8.10%	3.17%	8.50%
E2	0.00425	0	0	0.00%	0.00%	0.00%	0.00%	0.00%	0.43%	0.00332	0.01664	0	0	0	0.00%	0.00%	0.00%	0.00%
E3	8.05E-04	2.00E-06	6.53E-05	5.66E-05	5.72E-05	5.32E-05	5.20E-05	5.69E-05	0.14%	1.49E-04	2.00E-06	5.00E-05	6.53E-05	8.16E-05	9.89E-05	1.46E-04	1.89E-04	1.70E-04
TC	2.34E-04	2.75E-07	0	0.00%	0.00%	0.00%	0.00%	0.00%	2.34E-04	1.04E-04	4.97E-05	2.77E-05	3.62E-05	4.53E-05	5.95E-05	1.12E-04	8.95E-05	9.45E-05
OTC	3.94E-04	0	5.52E-05	8.73E-05	5.43E-05	0.00%	0.00%	0.00%	6.49E-04	3.96E-04	9.65E-06	5.16E-04	3.83E-04	3.33E-04	2.33E-04	2.13E-04	2.16E-04	1.90E-04
CTC	8.76	1.74	0.00	0.43	6.37	2.20	7.81	1.30	1.63	2.06	5.42	0.00	0.00	8.09	8.87E	6.66	9.08	8.50

	E-04	E-04	287	%	E-04	E-04	E-04	E-04	%	E-04	E-06	165	301	E-04	-04	E-04	E-04	E-04
∑EDCs+∑antibiotics	3.3028599	40.5280007	0.0156738	0.022797152	0.03219788	0.01588979	0.025727	0.0159596	3.6028434	0.0793655	0.0198504	0.043304	0.039424	0.0855389	0.0320687	0.089047	0.03385829	0.08765454
Algae																		
	Wet									Dry								
	Influent	Effluent	S1	S2	S3	S4	S5	S6	S7	Influent	Effluent	S1	S2	S3	S4	S5	S6	S7
BPA	1.15211	0.00345	8.97E-04	0.11%	9.16E-04	5.45E-04	4.81E-04	4.19E-04	3.65E-04	0.02223	5.68E-04	0.05573	0.04496	0.03959	3.79%	3.48%	0.40%	0.66%
NP	2.34818	0.11239	0.0133	0.96%	1.39%	2.40%	1.19%	1.90%	1.20%	0.05418	0.00232	0.02291	0.02064	0.05848	1.78%	6.20%	2.43%	6.51%
E2	2.17E-04	0	0	0.00%	0.00%	0.00%	0.00%	0.00%	0.00%	1.70E-04	0	0	0	0	0.00%	0.00%	0.00%	0.00%
E3	2.41E-04	5.99E-07	6.23E-05	1.96E-05	1.69E-05	1.71E-05	1.59E-05	1.56E-05	1.70E-05	4.46E-05	5.99E-07	1.50E-05	1.96E-05	2.44E-05	2.96E-05	4.38E-05	5.65E-05	5.10E-05
TC	0.06565	7.73E-05	0	0.00%	0.00%	0.00%	0.00%	0.00%	0.00%	0.02921	0.01395	0.00778	0.01016	0.0127	1.67%	3.14%	2.51%	2.65%
OTC	0.09912	0	0.01459	1.39%	2.20%	1.37%	0.00%	0.00%	0.00%	0.09965	0.00243	0.12982	0.09623	0.08383	5.86%	5.35%	5.42%	4.77%
CTC	0.04	0.00	0.31	14.18	21.28	3.15	1.09	3.86	0.64	0.01	2.68	0.08	0.14	0.03	4.38	3.29	4.48	4.20

	326	859	184	%	%	%	%	%	%	017	E-04	134	867	992	%	%	%	%
∑EDCs+∑antibiotics	3.7087776	3.3028599	0.3406891	0.166389551	0.24957308	0.06967229	0.023267	0.0580049	0.0188116	0.2156541	0.0195361	0.297595	0.32068	0.2345444	0.174799611	0.214534	0.15244653	0.18790096

References

1. Lee C-C, Jiang L-Y, Kuo Y-L, Chen C-Y, Hsieh C-Y, Hung C-F, Tien C-J: Characteristics of nonylphenol and bisphenol A accumulation by fish and implications for ecological and human health. *Science of The Total Environment* 2015, 502:417-425.
2. Wright-Walters M, Volz C, Talbott E, Davis D: An updated weight of evidence approach to the aquatic hazard assessment of Bisphenol A and the derivation a new predicted no effect concentration (Pnec) using a non-parametric methodology. *Science of The Total Environment* 2011, 409(4):676-685.
3. Jiang R, Liu J, Huang B, Wang X, Luan T, Yuan K: Assessment of the potential ecological risk of residual endocrine-disrupting chemicals from wastewater treatment plants. *Science of The Total Environment* 2020, 714:136689.
4. Lu S, Lin C, Lei K, Xin M, Wang B, Ouyang W, Liu X, He M: Endocrine-disrupting chemicals in a typical urbanized bay of Yellow Sea, China: Distribution, risk assessment, and identification of priority pollutants. *Environmental Pollution* 2021, 287:117588.
5. Czarny K, Szczukocki D, Krawczyk B, Skrzypek S, Zieliński M, Gadzała-Kopciuch R: Toxic effects of single animal hormones and their mixtures on the growth of Chlorella vulgaris and Scenedesmus armatus. *Chemosphere* 2019, 224:93-102.
6. Wang Q, Xu H, Gan S, Sun R, Zheng Y, Craig NJ, Sheng W, Li J-Y: Antibiotics and endocrine disrupting chemicals in effluent from wastewater treatment plants of a mega-city affected the water quality of juvenile Chinese sturgeon habitat: Upgrades to wastewater treatment processes are needed. *Marine Pollution Bulletin* 2025, 215:117840.
